# Optimizing thiopurine therapy in children with acute lymphoblastic leukemia: A promising “MINT” sequencing strategy and therapeutic “DNA-TG” monitoring

**DOI:** 10.3389/fphar.2022.941182

**Published:** 2022-09-27

**Authors:** Hong-Li Guo, Yue-Tao Zhao, Wei-Jun Wang, Na Dong, Ya-Hui Hu, Yuan-Yuan Zhang, Feng Chen, Li Zhou, Tao Li

**Affiliations:** ^1^ Pharmaceutical Sciences Research Center, Department of Pharmacy, Children’s Hospital of Nanjing Medical University, Nanjing, China; ^2^ School of Basic Medicine and Clinical Pharmacy, China Pharmaceutical University, Nanjing, China; ^3^ Visiting Graduate Student from School of Basic Medicine and Clinical Pharmacy, Pharmaceutical University, Nanjing, China; ^4^ Institute of Pharmaceutical Science, China Pharmaceutical University, Nanjing, China; ^5^ School of Institute of Pharmaceutical Science, Pharmaceutical University, Nanjing, China; ^6^ Hematology and Oncology Department, Children’s Hospital of Nanjing Medical University, Nanjing, China; ^7^ Department of Solid Oncology, Children’s Hospital of Nanjing Medical University, Nanjing, China

**Keywords:** thiopurines, myelosuppression, pharmacogenetics, “MINT” sequencing strategy, therapeutic “DNA-TG” monitoring

## Abstract

Thiopurines, including thioguanine (TG), 6-mercaptopurine (6-MP), and azathioprine (AZA), are extensively used in clinical practice in children with acute lymphoblastic leukemia (ALL) and inflammatory bowel diseases. However, the common adverse effects caused by myelosuppression and hepatotoxicity limit their application. Metabolizing enzymes such as thiopurine S-methyltransferase (TPMT), nudix hydrolase 15 (NUDT15), inosine triphosphate pyrophosphohydrolase (ITPA), and drug transporters like multidrug resistance-associated protein 4 (MRP4) have been reported to mediate the metabolism and transportation of thiopurine drugs. Hence, the single nucleotide polymorphisms (SNPs) in those genes could theoretically affect the pharmacokinetics and pharmacological effects of these drugs, and might also become one of the determinants of clinical efficacy and adverse effects. Moreover, long-term clinical practices have confirmed that thiopurine-related adverse reactions are associated with the systemic concentrations of their active metabolites. In this review, we mainly summarized the pharmacogenetic studies of thiopurine drugs. We also evaluated the therapeutic drug monitoring (TDM) research studies and focused on those active metabolites, hoping to continuously improve monitoring strategies for thiopurine therapy to maximize therapeutic efficacy and minimize the adverse effects or toxicity. We proposed that tailoring thiopurine dosing based on *MRP4*, *ITPA*, *NUDT15*, and *TMPT* genotypes, defined as “MINT” panel sequencing strategy, might contribute toward improving the efficacy and safety of thiopurines. Moreover, the DNA-incorporated thioguanine nucleotide (DNA-TG) metabolite level was more suitable for red cell 6-thioguanine nucleotide (6-TGNs) monitoring, which can better predict the efficacy and safety of thiopurines. Integrating the panel “MINT” sequencing strategy with therapeutic “DNA-TG” monitoring would offer a new insight into the precision thiopurine therapy for pediatric acute lymphoblastic leukemia patients.

## Introduction

Thioguanine (TG), 6-mercaptopurine (6-MP), and azathioprine (AZA), collectively known as thiopurines, are broadly applied as anti-cancer and immunosuppressive agents. 6-MP is one of the backbone drugs for the maintenance therapy of acute lymphoblastic leukemia (ALL) in children and adults (Schmiegelow et al., 2014). 6-MP and AZA are also commonly prescribed in maintaining the clinical remission of patients with steroid-dependent inflammatory bowel diseases (IBD) (Andoh et al., 2021). Thiopurines are pro-drugs devoid of any intrinsic activity and need metabolic transformation to their pharmacologically active metabolites, 6-thio-guanine nucleotides (6-TGNs), which are structurally similar to the endogenous purine-base guanine, are fraudulent bases being integrated into the DNA of leucocytes, leading to the inhibition of purine *de novo* synthesis and cell death (Karran and Attard, 2008). It is worth noting that, while thioguanine enters the DNA to play a therapeutic role, it also creates conditions for the occurrence of common and serious adverse reactions. Hepatotoxicity, pancreatitis, gastric intolerance, and leukopenia are common adverse drug reactions associated with thiopurines (Koren et al., 1990; Lee et al., 2022). In particular, life-threatening leukopenia, caused by thiopurines, might interrupt or even discontinue the effective treatment, resulting in a high risk of subsequent disease recurrence in ALL (Fotoohi et al., 2010). Hence, the narrow therapeutic index of thiopurines indicates an urgent demand for us to utilize precision medicine strategies to make better use of these drugs.

Polymorphisms of genes encoding various drug-metabolizing enzymes and transporters could exert an effect on the efficacy and toxicity of thiopurines. In fact, the first pharmacogenetic marker by means of a pharmacology-guided approach was recognized as single nucleotide polymorphisms (SNPs) in thiopurine S-methyltransferase (TPMT) (Weinshilboum and Sladek, 1980), and its predictive role in 6-MP-related adverse effects is regarded as an important development in this field. In recent years, other related enzymes including Nudix hydrolase 15 (NUDT15) (Yang et al., 2014), inosine triphosphate pyrophosphohydrolase (ITPA) (Barba et al., 2022), and multidrug resistance-associated protein (e.g., MRP4) (Wielinga et al., 2002) have been reported to dramatically affect the pharmacokinetics and pharmacological properties of thiopurines, thereby becoming critical determinants of their therapeutic efficacy and toxicity. However, the significant differences in the distribution and frequency of these SNPs among different ethnic groups pose certain challenges for a practical clinical application of genotype-based precision therapy.

Previously, monitoring the drug concentration of thioguanine nucleotides (TGNs) in red blood cells (RBCs) has played an important role in maximizing the clinical efficacy and reducing adverse effects of these drugs, and has also been helpful for individualized dose adjustment (Nygaard et al., 2004; Hedeland et al., 2010; Nielsen et al., 2016). Unfortunately, there is a broad agreement that too high or too low TGN concentrations should be avoided, but the exact definition of both upper and lower limits, even if such rigid threshold values should be generally used at all, is still debated in different studies. Furthermore, as RBCs are not the active target of thiopurines, some researchers proposed that the cytotoxicity of thiopurines was caused by the incorporation of thioguanine nucleotides into the DNA, called DNA-TG, the true culprit in the nucleus. The concentration of DNA-TG has been quantified by using liquid chromatography–mass spectrometry (Moriyama et al., 2017; Larsen et al., 2021a). Therefore, DNA-TG monitoring is much more helpful in evaluating an inadequate dose or non-adherence, or distinguishing patients with abnormal metabolic spectrums (Nielsen et al., 2017).

In this review, we summarize the pharmacogenetic studies related to those enzymes and transporters taking responsibility for the disposition of thiopurines, focusing on the association between SNPs and the clinical efficacy and side effects of thiopurines. We also review the current status of the TDM of thiopurines in children with ALL. Combining “MINT” panel sequencing with DNA-TG monitoring is a more feasible strategy when implementing thiopurine precision medicine for those pediatric patients.

## Metabolism and material action basis of thiopurines

**FIGURE 1:**
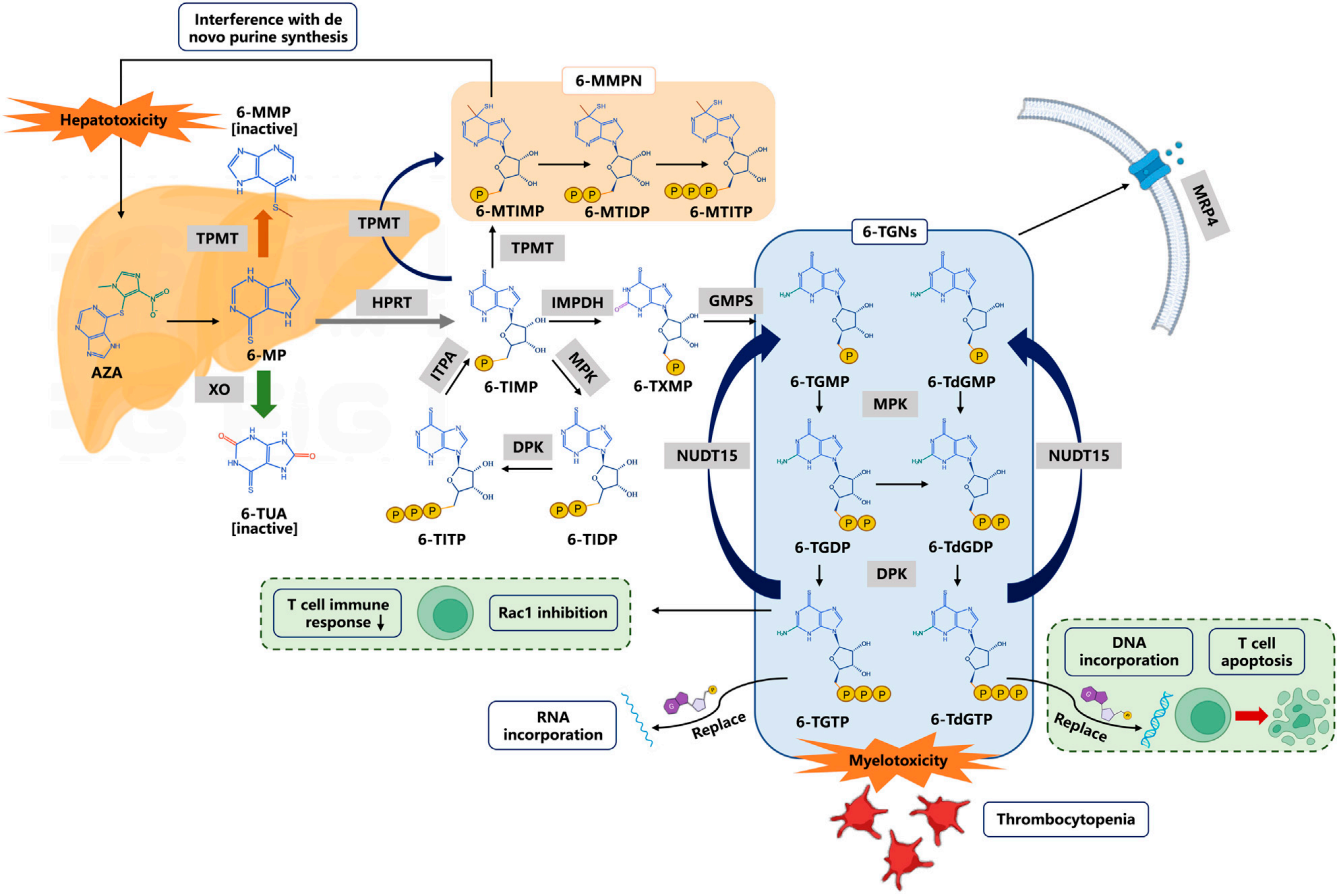
Thiopurine metabolism and transportation. 6-Thioguanine nucleotides (6-TGNs) are pharmacologic active products. 6-TGMP 6-thioguanine monophosphate, 6-TGDP 6-thioguanine diphosphate, 6-TGTP 6-thioguanine triphosphate, 6-TdGMP 6-thio-deoxyguanine monophosphate, 6-TdGDP 6-thio-deoxyguanine diphosphate, 6-TdGTP 6-thio-deoxyguanine triphosphate. Abbreviations: AZA azathioprine, 6-MP 6-mercaptopurine, 6-MMP 6-methylmercaptopurine, 6-TUA 6-thiouric acid, 6-TIMP 6-thioinosine monophosphate, 6-MMPN 6-methylmercaptopurine nucleotides, 6-MTIMP 6-methylthioinosine 5′-monophosphate, 6-MTIDP 6-methylthioinosine 5’ -diphosphate, 6-MTITP 6-methylthioinosine 5′-triphosphate, 6-TIDP 6-thioinosine diphosphate, 6-TITP 6-thioinosine triphosphate, 6-TXMP 6-thioxanthosine monophosphate. Enzymes or transporters are shown in gray boxes: TPMT thiopurine S-methyl transferase, XO xanthine oxidase, HPRT hypoxanthine phosphoribosyl transferase, MPK monophosphate kinase, DPK diphosphate kinase, ITPA inosine triphosphate pyrophosphohydrolase, IMPDH inosine monophosphate dehydrogenase, GMPS guanosine monophosphate synthetase, and MRP4 multidrug resistance-associated protein 4.

As prodrugs that lack intrinsic activity or with low activity, thiopurines need to be transformed to 6-TGNs inside the cells through multi-enzymatic reactions and then exert pharmacological effects. The three major metabolic pathways of 6-MP are well described (Figure 1). Xanthine oxidase (XO) and thiopurine-S-methyltransferase (TPMT) are the two dominant enzymes in the metabolism of 6-MP, and produce inactive metabolites 6-thiouric acid and 6-methylmercaptopurine (6-MMP), respectively. However, the formation of 6-TGNs is a “long journey” involving multiple enzymes. 6-MP is first converted into thioIMP (6-TIMP) by hypoxanthine-guanine phosphoribosyltransferase (HGPRT), and finally, 6-TIMP metabolized to 6-TGNs, the active forms, which are successively mediated by hypoxanthine monophosphate dehydrogenase (IMPDH) and guanylate synthase (GMPS), respectively. Meanwhile, the multidrug resistance protein 4 (MRP4) can pump these active metabolites out of the cells. In this journey, TPMT can also metabolize intermediate 6-TIMP into methyl-thioIMP (6-MTIMP), an active metabolite that can suppress *de novo* purine synthesis, and has been reported to have an association with hepatotoxicity (Adam de Beaumais et al., 2011). 6-TIMP also can be catalyzed into 6-thioinosine diphosphate (6-TIDP) by monophosphate kinase (MPK), and into 6-thioinosine triphosphate (6-TITP) by diphosphate kinase (DPK), and finally back to 6-TIMP by ITPA forming a complete cycle.

6-TGNs are active metabolites, consisting of 6-thioguanine monophosphate (6-TGMP), 6-thio-deoxyguanine monophosphate (6-TdGMP), 6-thioguanine diphosphate (6-TGDP), 6-thio-deoxyguanine diphosphate (6-TdGDP), 6-thioguanine triphosphate (6-TGTP), and 6-thio-deoxyguanine triphosphate (6-TdGTP). The aforementioned 6 metabolites are catalyzed by MPK, DPK, and NUDT15, respectively, to form another metabolic cycle. On one hand, 6-TdGTPs are integrated into DNA (6-TdGTP) and RNA (6-TGTP), leading to nucleotide suppression and protein synthesis and cause cell apoptosis (Paugh et al., 2010). On the other hand, 6-TGTPs have been reported to block the activity of Vav on RAC proteins and thus to prevent the development of an effective immune response in T cells (Poppe et al., 2006). It is generally accepted that the adverse reactions of thiopurines are closely associated with those active metabolites, especially high levels of 6-TGNs resulting in dose-dependent side effects typified by leukopenia (Relling et al., 1999). Therefore, the genetic variation of key enzymes in metabolic transformations (such as TPMT, ITPA, and NUDT15) would have an important impact on the pharmacokinetics, pharmacodynamics, and side effects of thiopurines.

## Pharmacogenetics of thiopurines

### Thiopurine S-methyltransferase

As one of the most critical enzymes in the biotransformation of thiopurines, TPMT is the earliest and most comprehensive pharmacogenetic predictor used in clinical practice. Loss of the TPMT function leads to excessive levels of 6-TGNs, which greatly increase the risk of leukopenia. In 1980, Weinshiboum and Sladek first reported the significant individual variations of erythrocyte TPMT activity in Caucasians, with 88.6% of high, 11.1% of intermediate, and 0.3% of undetectable activities, respectively (Weinshilboum and Sladek, 1980). Since then, this classification method has been widely used. Moreover, SNPs resulting in loss of function of the TPMT have also been identified (Ansari et al., 2002). More than 40 allelic variants (*2–*44) in the *TPMT* gene have been discovered (Iu et al., 2017; Zimdahl Kahlin et al., 2019), but the loss-of-function variants mainly include TPMT*2, TPMT*3A, TPMT*3B, and TPMT*3C (Azimi et al., 2014). Therefore, the TPMT phenotype is usually determined by the aforementioned mutant variants. Especially, *TPMT* genotypes *3A and *3C had the strongest associations with toxicity (Rudin et al., 2017). In 2011, the Clinical Pharmacogenetics Implementation Consortium (CPIC) guidelines for *TPMT* genotype and thiopurine dosing (Relling et al., 2011) recommend pre-emptive genotyping for *TPMT* before the initiation of thiopurine treatment, and that patients with heterozygous *1 allele and with *2/*3A/*3C/*4 alleles (intermediate metabolizers) should start at 30–70% of the full dose (6-MP 50 mg/m^2^/day or 0.75 mg/kg/day). However, for poor metabolizers, the thiopurine dose should be purposefully started with 10% of the full dose, and administered weekly thrice instead of daily. This approach has been proven to decrease the incidence of acute toxicity while having no negative effect on the relapse rates in ALL (Relling et al., 2006).

Despite the success of *TPMT* pharmacogenetic testing, the most important issue is that the prevalence of loss-of-function *TPMT* alleles varies across ethnic groups (Table 1, https://www.pharmgkb.org). In Europeans and African Americans, the common alleles that cause TPMT deficiency are TPMT*2, *3A, *3B, and *3C, while in Asians, *3C is the most common mutant allele. A recent extensive whole-genome re-sequencing study in 3,554 Japanese showed that alleles *3A and *3B were absent (not observed), and only allele *3C was confirmed to be present (0.96%) (Nagasaki et al., 2015; Yamaguchi-Kabata et al., 2015). Furthermore, in a study including 253 Chinese patients, none of the three alleles *2, *3A, and *3B were observed, and the allelic frequency of *3C was 1.6% (Zhu et al., 2016). In this study, leukopenia occurred in 25.7% of patients, and no strong association was observed between the disease and *3C genotypes. Clearly, the low-mutation frequency (<2%) of variants in the *TPMT* gene did not explain the high frequency of adverse reactions (>20%) occurring in East Asian populations, suggesting that pre-emptive *TPMT* genotyping in Asians might not be the same as that in Caucasians. On the other hand, there are a plethora of genetic variants (*1∼*44) of *TPMT* as mentioned previously, and it is hard to determine a patient’s exact genotype solely based on one commercial kit which usually cannot provide all target variations. Moreover, other genetic variants may contribute to inter-patient variability. Hopefully, those problems would be readily solved as testing costs are reduced and pharmacogenetic implementations become very popular.

**TABLE 1:** Frequencies of the TPMT and NUDT15 alleles/phenotypes in major race/ethnic groups.

—	Allele	African	Central/South Asian	East Asian	European	Phenotype	African	Central/South Asian	East Asian	European
TPMT	*1	92.34%	98.14%	97.96%	95.31%	NM	85.27%	96.31%	95.97%	90.84%
	*2	0.53%	0.02%	0.01%	0.21%	IM	6.89%	3.41%	3.34%	8.42%
	*3A	0.80%	0.42%	0.03%	3.43%	PIM	0.29%	0.00%	0.01%	0.02%
	*3B	0.00%	0.17%	0.00%	0.27%	PM	0.14%	0.03%	0.03%	0.20%
	*3C	2.40%	1.12%	1.64%	0.47%	Indeterminate	7.41%	0.24%	0.65%	0.53%
NUDT15	*1	99.69%	93.00%	87.90%	99.31%	NM	99.38%	86.49%	77.26%	0.53%
	*2	0.00%	14.3%	3.50%	0.00%	IM	0.27%	12.55%	16.79%	8.42%
	*3	0.10%	6.70%	6.05%	0.20%	PIM	0.00%	0.03%	0.49%	90.84%
	*4	0.03%	0.00%	0.09%	0.00%	PM	0.00%	0.46%	0.91%	0.20%
	*5	0.00%	0.04%	1.11%	0.00%	Indeterminate	0.35%	0.46%	4.55%	0.02%
	*6	0.15%	0.20%	1.30%	0.30%					

NM: Normal metabolizer; IM: Intermediate metabolizer; PIM: Possible intermediate metabolizer; PM: Poor metabolizer.

Data from PharmGKB (https://www.pharmgkb.org) and study of Banerjee R and colleagues (Banerjee et al., 2020).

Another concern is that results from a pharmacogenetic test are incapable of covering the other co-factors contributing to the TPMT phenotype such as age, renal insufficiency, or drug–drug interactions (Wu et al., 2019). From this perspective, measuring TPMT activity is a more accurate strategy for predicting the appropriate dose of thiopurines than genotyping test. Actually, TPMT phenotype testing is fairly common in some countries (Fargher et al., 2007; Weitzel et al., 2018). However, it is worth noting that TPMT enzyme activity is usually measured in fresh red blood cells directly (Mokhtari et al., 2020), or consumes a longer turnaround test time (Weitzel et al., 2018). Moreover, in patients who have received blood transfusions recently or in leukemia patients, because of atypical hematopoiesis, the result could not reflect the true enzymatic activity (Mokhtari et al., 2020).

Interestingly, genotype–phenotype discordance was reported at a rate of 5% in patients who underwent both tests (Weitzel et al., 2018). In patients with a discordant situation, the use of genotypes to guide thiopurine dosing is consistent with a previous study that states that optimum accuracy of the *TPMT* genotype test was achieved as compared with the enzyme activity assay (Donnan et al., 2011).

### Nucleoside diphosphate-linked moiety X-type motif 15

In 2014, Yang and others first reported that *NUDT15* (rs116855232, referred to as c.415C > T or p.R139C variant hereafter) was highly associated with thiopurine-induced leukopenia among Korean patients who suffered from IBD (Yang et al., 2014). Subsequently, loss-of-function *NUDT15* diplotypes were found to be consistently related to the intolerance of thiopurine during ALL therapy (Yang et al., 2015; Moriyama et al., 2016). Based on these studies, CPIC in 2018 updated its guidelines for the dosage adjustment of 6-MP, suggesting that ALL patients should be guided by *TPMT* and *NUDT15* genotyping for the initial dose selection of 6-MP (Relling et al., 2019).

NUDT15 is a purine-specific nudix hydrolase that controls the hydrolysis of nucleosides–diphosphates (Gad et al., 2014). This enzyme catalyzes the conversion of TGTP and deoxy-TGTP (TdGTP) metabolites to the low-toxic TGMP and deoxy-TGMP (TdGMP), respectively (Valerie et al., 2016). Defective NUDT15-mediated degradation of TdGTP results in more available TdGTP which can be used for the integration into the DNA (namely DNA-TG, the primary anti-leukemic metabolite). Simultaneously, NUDT15 deficiency also results in more TGTP, which promotes the binding of TGTP to Rac1 and also promotes the transportation of TGTP into the RNA. Therefore, it is hypothesized that NUDT15 negatively regulates thiopurine activation and its cytotoxicity (Moriyama et al., 2016). Although 6-MP-induced leukopenia is known to be related to increased 6-TGNs levels, no significant difference was found between the 6-TGN levels and *NUDT15* variants (Asada et al., 2016). Interestingly, results obtained from a series of model systems in a Moriyama laboratory and ALL patients jointly indicated that NUDT15 deficiency directly led to the excessive DNA-TG levels and increased adverse effects (Moriyama et al., 2016). Therefore, the DNA-TG metabolite levels are preferred over 6-TGNs in forming *NUDT15* genotype-guided dose adjustments (Moriyama et al., 2017).

As the first *NUDT15* SNP linked to thiopurine toxicity, p.R139C was the most studied in patients receiving thiopurine therapy, and the results showed that *NUDT15* p.R139C mutation had no influence on the enzymatic activity. Instead, it negatively affected the protein stability (Valerie et al., 2016). In children diagnosed with ALL, the tolerability of homozygotes for the p.R139C variant allele was only 8% of the standard dose of 6-MP, while the tolerable dosages were 63 and 83.5% of the standard doses of 6-MP, respectively, in those patients with heterozygous and wild-type genotypes (Yang et al., 2015). To date, a total of 20 haplotypes with star allele names of *NUDT15* (*1-*20) have been identified (Moriyama et al., 2016; Moriyama et al., 2017; Schaeffeler et al., 2019) (https://www.pharmvar.org/gene/NUDT15). It was found that in Chinese IBD patients, the predictive sensitivity of *NUDT15* p.R139C was 49.2 %. But, the combined analysis of Val18Ile and p.Val18_Val19insGlyVal, designed to identify diplotypes by detecting haplotypes *5 and *6, successfully increased the sensitivity to 55.4 % (Chao et al., 2017). However, compared with p.R139C, other variants are rare, and their correlations with thiopurine-induced toxicity in clinical practice remain unclear. As shown in Table 1, the frequency of the decreased function NUDT15 variants is higher in Asians and Hispanics, but lower in Europeans and Africans. Interestingly, the frequency is just reverse for *TPMT* variants. Therefore, as the CPIC guideline recommended, if genetic tests are available for only one gene (*TPMT* or *NUDT15*, but not both), the matched decision-making will be implemented by clinical labs. More importantly, the potential association of rare mutations with efficacy and adverse reactions of thiopurines remains to be confirmed by large cohort studies.

### Inosine triphosphate pyrophosphohydrolase

ITPA can catalyze the phosphorylation of inosine triphosphate (ITP) and convert the latter to inosine monophosphate (IMP), which is a key substance in the purine metabolism. In the metabolism of 6-MP, ITPA is the catalyst that helps to complete the hydrolysis process from 6-TITP to 6-TIMP, and the ITPA deficiency leads to an abnormal accumulation of 6-TITP, which results in toxicity (Marinaki et al., 2004). There are 11 *ITPA* variants that have been reported (Sakamoto et al., 2020), and the two most common SNPs are 94C > A as well as IVS2 + 21A > C. Several studies have examined the role of these two variants in the *ITPA* gene with 6-MP metabolism, as well as adverse drug reactions including hepatotoxicity, flu-like symptoms, arthralgia, and pancreatitis (Arenas et al., 2007; Zabala-Fernandez et al., 2011; Wroblova et al., 2012) with promising results. However, consistent with *TPMT* polymorphism, the frequency of the *ITPA* (94C > A) A allele differed significantly by ethnicity and was higher in Asians (11–19%) than in Caucasian, Hispanics, and Africans (1–7%) (Okada et al., 2009). Interestingly, the reverse situation would appear for the frequency of the *ITPA* 94C > A allele and *TPMT* polymorphisms in the same populations (Marsh and Van Booven, 2009).

Naturally, research studies on the relationship between the *ITPA* gene polymorphism and the adverse reactions of thiopurines are inconsistent partly due to the ethnic differences in allele frequencies (Table 2). For instance, Uchiyama found that the *ITPA* 94C > A mutation occurred more frequently than *TPMT* variants in Japanese patients diagnosed with thiopurine-induced leukopenia (Uchiyama et al., 2009). Also, ALL patients with allele *ITPA* 94A were more likely to suffer from fever and hepatotoxicity from 6-MP, while the prevalence of *TPMT* variants was too low to be well applied in Malays, Chinese, and Indian populations (Wan Rosalina et al., 2012). Therefore, Asians may be more sensitive to the toxicity of AZA/6-MP based on the *ITPA* mutation than *TPMT*. However, a lack of association between the *ITPA* 94C > A polymorphism and AZA-related adverse effects was found in a New Zealander (Gearry et al., 2004). In addition, *ITPA* genotyping has no predictive significance for the clarity and development of the AZA side effects (van Dieren et al., 2005). In the Netherlands, the mean doses of 6-MP did not differ in ALL patients with or without *ITPA* variants (Kouwenberg et al., 2020). Another study came to a similar conclusion (Wahlund et al., 2020). The aforementioned results suggested that, before the beginning of maintenance treatment for ALL in these populations, the *ITPA* genotype should not to be regarded as a part of the accepted assessment.

**TABLE 2:** Frequencies of the ITPA and MRP4 variants in major race/ethnic groups.

—	Allele	African	Latino	East Asian	European	South Asian
ITPA	rs1127354 (C > A/G)	4.46%	4.18%	16.87%	7.06%	12.17%
	rs7270101 (A > C)	7.11%	8.21%		12.92%	1.53%
MRP4	rs3765534 (C > T)	0.08%	2.74%	7.64%	0.89%	5.11%
	rs2274407 (C > A/G/T)	20.95%	6.20%	18.45%	8.05%	17.08%

Data from PharmGKB (https://www.pharmgkb.org).

Stocco and others analyzed a group of St. Jude patients with ALL whose 6-MP doses were not adjusted based on their *TPMT* genotype or TGN concentrations. Notably, the probability of grade 3–4 infections was not significantly related to the *ITPA* genotype in the aforementioned conditions. But if the doses were tailored for *TPMT* and *ITPA*, then they had a great effect on the likelihood of febrile neutropenia (Stocco et al., 2010). According to the authors, most studies suggested that the dose of 6-MP taken by patients was not fully adjusted for the *TPMT* genotype. Their results revealed that it might be the cause of why the influence of the *ITPA* genotype had been inconsistent in previous studies. Some researchers tend to suggest that physicians should first ponder over the genotyping for *ITPA* variants, together with *TPMT* and *NUDT15*, before deciding to treat a patient with 6-MP (Moradveisi et al., 2019). Of note, the association between *ITPA* 94 C > A and neutropenia in children with ALL was verified in recent systematic reviews and meta-analysis (Barba et al., 2022; Lee et al., 2022). A total of 1,072 and 974 ALL pediatric patients were included in the meta-analysis, respectively. Specifically, pediatric ALL patients with an *ITPA* 94 C > A variant had an approximately 2.5 times higher risk of suffering from neutropenia (Barba et al., 2022; Lee et al., 2022). Moreover, due to the existing ethnic differences in the *ITPA* 94 C > A mutation frequency, both the studies had stratified their data analysis based on the races. For neutropenia, the results did not show any different outcome between Asians and Caucasians (Barba et al., 2022), while for hepatotoxicity, the 94C > A variant was significantly associated with an increased risk in Asians and Middle Easterners (Lee et al., 2022).Moreover, IBD patients with *ITPA* variant alleles exhibited higher 6-TGN levels than those with the wild-type allele (Luo et al., 2022). These findings support that *ITPA* polymorphisms could be used as predictive biomarkers for thiopurine-related adverse effects. Nevertheless, considering the variable frequency across different ethnicities, the clinical implementation of *ITPA* gene tests for precision thiopurine treatment also warrants further studies.

### Multi-drug resistance protein 4

MRP4 is a member of the ATP-binding cassette transporter family, encoded by the *ABCC4* gene, responsible for transporting monophosphorylated nucleosides (Wielinga et al., 2002). Murine models with MRP4 deficiency confirmed that MRP4 protects against thiopurine-induced hematopoietic toxicity by reducing the accumulation of intracellular TGNs (Takenaka et al., 2007; Krishnamurthy et al., 2008). In clinics, Hiromistu Ban and others were the first to demonstrate an association of *MRP4* G2269A (rs3765534) polymorphisms with thiopurine sensitivity (Ban et al., 2010). The authors found that patients with the *MRP4* variant had higher 6-TGN levels in erythrocytes and a higher risk of leukopenia, compared with patients with the wild-type alleles. In addition, another study highlighted the significance of *MRP4* polymorphisms related to 6-MP dose tolerance in ALL maintenance therapy (Tanaka et al., 2015). Moreover, ALL patients with intermediate active *NUDT15* and *ABCC4* variants experienced higher 6-MP intolerability, compared with the group with either of the variants (Tanaka et al., 2018). In a very recent study, the co-occurrence of the *ABCC4* (c.912G > T, rs2274407) and *ITPA* (c.94C > A) variants in 145 Chinese children with ALL witnessed a significant positive association with 6-MP intolerance (Fan et al., 2022). Meanwhile, *ABCC4* c.2128G > A (rs3765534) carriers experienced a significant increase in the DNA-TG to 6-MP dose ratio, which was associated with a high risk of leukopenia (Fan et al., 2022). Similarly, *ABCC4* SNP rs2274407 was found to be related to the increased 6-TGN to 6-MP dose ratio (Choi et al., 2019). In addition, a study in Thai ALL pediatric patients found that the average absolute neutrophil count (ANC) at the 6^th^ month of the maintenance phase was significantly lower in *ABCC4* SNP rs3765534 carriers, compared to patients carrying wild-type alleles, and the risk of grade 4 neutropenia was higher in *ABCC4* GA carriers than wild-type patients, but was not statistically significant (Khaeso et al., 2022). Of note, the allele frequency of variants in *ABCC4* showed ethnic difference (Table 2), and more evidence needs to be accumulated to establish the potential association between *ABCC4* variants and 6-MP-induced adverse effects in the future.

### Other genetic polymorphisms

In 2012, Stocco’s team first reported that *PACSIN2* (protein kinase C and casein kinase II interacting protein-2 or Syndapin 2) was the most fundamental trans-acting gene. *PACSIN2* has also been shown to be a genetic variant which could affect TPMT activity (Stocco et al., 2012). Moreover, a SNP of *PACSIN2* (rs2413739) was closely associated with severe gastrointestinal toxicity in children with ALL treated with 6-MP. Recently, another study confirmed these results (Franca et al., 2020). Furthermore, *PACSIN2* polymorphism was shown to be linked with thiopurine-induced hematological toxicity in children receiving maintenance therapy aimed at treating ALL (Smid et al., 2016). As an earlier study had identified, PACSIN2 was a Rac1 interactor that regulated the diffusion of Rac1-mediated cells (de Kreuk et al., 2011). Therefore, the interaction of PACSIN2 with Rac1 might increase the likelihood of hematotoxicity, resulting in increased sensitivity of cells to 6-MP, thereby exposing patients to a higher risk of hematotoxicity.

Cytosolic 5′-nucleotidase II (NT5C2) is an allosteric catabolic enzyme that hydrolyzes IMP, GMP, and AMP. The thiopurine nucleotides thio-GMP, thio-IMP, and methylthioIMP are all converted by NT5C2 (Brouwer et al., 2005). Increased *in vitro* nucleotidase activity has been identified in NT5C2 mutant proteins. Also, when expressed in ALL lymphoblasts, these proteins were resistant to the chemotherapy with 6-MP/6-TG (Tzoneva et al., 2013). Tulstrup and others found that the thiopurine metabolism can be altered by *NT5C2*-germline variants associated with acquired recurrent *NT5C2* mutations in childhood acute lymphoblastic leukemia (Tulstrup et al., 2018). Moreover, sub-clonal *NT5C2* mutations determine relapses related to the high risks in treatment failure in patients, while emphasizing their complicated role in the outcome over mutant *NT5C2*, which acts as a targetable driver during relapse progression. Therefore, a thorough, rigorous, and retrospective study is warranted so that we could identify *NT5C2* mutations, further deepening our understanding and better treating the relapse subtype of aggressive ALL (Barz et al., 2020).

Recently, folate metabolic variants including thymidylate synthetase (*TYMS*) and dihydrofolate reductase (*DHFR*) were shown to be potential biomarkers of 6-MP-induced myelotoxicity, which could be employed for the individualization of 6-MP therapy when childhood ALL treatment reaches its maintenance phase (Milosevic et al., 2019). In Chinese pediatric patients, the *MTHFR* rs1801133 variant was found to have a 4.46-fold higher risk of hepatotoxicity than the wild-type genotype (Zhou et al., 2020). In this study, the authors also found that *IMPDH1* (rs2278293) was associated with a high risk of leukopenia (Zhou et al., 2020), which is consistent with Rihwa Choi’s earlier study in Korean ALL patients (Choi et al., 2019). Moreover, Rihwa Choi and others analyzed 103 SNPs and found that, in addition to the *TPMT* genotype, thiopurine metabolism and any adverse effects were linked with a total of 32 SNPs in 24 genes (Choi et al., 2019). A series of genetic polymorphisms such as *ADK*, *ATIC*, *GART*, *GMPS*, *GSTP1*, *SLC29A1*, *KCNMA1*, *SLC19A1*, *MOCOS*, *MTRR*, *SLC28A3*, *SLCO1B1*, and *XDH* have been linked with thiopurine-related adverse effects that have not been previously assessed.

Generally, the association between genotype and phenotype in pharmacogenetic studies is complex. As shown in Supplementary Table S1, we summarize the effects of *TMPT*, *NUDT15*, *ITPA*, and *MRP4* polymorphisms on the clinical outcomes of thiopurines in ALL pediatric patients. We propose that the ongoing research will surely manifest the value of pharmacogenetics in predicting the optimal dose and reducing adverse events, thereby contributing to better treatment for ALL patients.

## Therapeutic drug monitoring of thiopurines

The pharmacodynamics and pharmacogenetics of thiopurines have evolved considerably over the past 30 years. In addition to genotype–phenotype related research studies, long-term clinical practice has confirmed that thiopurine-associated adverse events and ALL relapses are related to metabolite levels, including 6-TGNs, 6-methylmercaptopurine nucleotides (6-MMPN), or the ratio of 6-MMPN/6-TGNs, and DNA-TG levels (Table 3). Therefore, integrating TDM of these active metabolites with pharmacogenetics may facilitate the development of a more personalized dosing method than the traditional weight-based one.

**TABLE 3:** Main efficacy, safety results, and TDM of thiopurines in ALL pediatric patients.

NO.	First author year	Study design and population	Treatment regimen and duration	Concomitant medication	Gene	Measured metabolites	Matrix	Metabolites range	Principal findings	Reference
1	Lennard 1983	Prospective, 22 patients, pediatric, European	6-MP 75 mg/m2/d > 2 weeks	Methotrexate Steroids Vincristine	—	6-TGN	RBC	0–802 (210 ± 149) (pmol/8 × 108 RBC)	As for the group not influenced by co-trimoxazole, as long as RBC 6-TG nucleotide at day 0 reaches >210 pmol/8 × 108 RBC, folate deficiency or neutropenia can be foreseen to happen by day 14	Lennard et al., (1983)
2	Lilleyman 1984	Prospective, 22 patients, pediatric, European	6-MP 75 mg/m2/d 11 months	Methotrexate Steroids Vincristine	—	6-TGN	RBC	girls: 0–720 boys: 42–958 (pmol/8 × 108 RBC)	For the girls involved in the study, statistically significant relevance between the doses of 6-MP and 6-TGN was shown, whereas this was not found in the boys	Lilleyman et al., (1984)
3	Lennard 1990	Retrospective, 95 patients, pediatric, European	6-MP 75 mg/m2/d > 2 months	Methotrexate Steroids Vincristine	TPMT	6-TGN	RBC	132–832 (pmol/8 × 108 RBC)	Among the 16 patients, 15 of them suffered a relapse, and this might be due to their lower 6-TGN concentrations, which did not attain the group median level. And this was a statistically significant excess, as it proved to be	Lennard et al., (1990)
4	Schmiegelow 1990	Prospective, 31 patients, pediatric, European	6-MP 50–75 mg/m2/d at least 3 months	Methotrexate	—	6-TGN	RBC	85–286 (nmol/mmol Hb)	Mean white cell count was used as an indicator to measure the degree of myelosuppression in this research and it was found that the degree of myelosuppression was correlated with 6-TGN.	Schmiegelow and Bruunshuus, (1990)
5	Berkovitch 1996	Retrospective, 25 patients, pediatric, North American	6-MP GI symptoms: 73 ± 23 mg/m2/d	Methotrexate Steroids Vincristine	—	6-MMPN	RBC	3000–27900 (pmol/8 × 108 RBC)	6-TGN as well as 6-MMPN levels in RBC, did not differ significantly in patients who had hepatotoxicity, when compared to those without hepatotoxicity	Berkovitch et al., (1996)
						6-TGN		171–463 (pmol/8 × 108 RBC)		
6	Lancaster 1998	Retrospective 23 patients, pediatric, European	6-MP 75 mg/m2/d > 12 weeks	Methotrexate Steroids Vincristine	—	6-TGN	RBC	205–1310 (pmol/8 × 108 RBC)	For participants who took TG on average, their 6-TGN levels were found to be 5-fold higher. At the same time, in the group of children on MP, 6-TGN levels had no obvious relevance to myelotoxicity, the results demonstrated	(Lancaster et al., (1998)
7	Chrzanowska 1999	Retrospective 19 patients, pediatric, European	6-MP 50 mg/m2/d > 1 month	Methotrexate	—	6-MMPN	RBC	<150–19000 (pmol/8 × 108 RBC)	There was a significant relevance observed between WBC count and RBC 6-TGN. Also, the same thing applies to neutrophil count and RBC 6-TGN.	Chrzanowska et al., (1999)
						6-TGN		<60–833 (pmol/8 × 108 RBC)		
8	Innocenti 2000	Retrospective 19 patients, pediatric, European	6-MP 50 mg/m2/d 3–18 months	Methotrexate Cytosine Steroids Vincristine	—	6-TGN	RBC	74–628 (pmol/8 × 108 RBC)	It was demonstrated to be significantly correlated that as RBC 6-TGN levels increased, the decrease of WBC, ANC, erythrocyte, and platelet counts was observed	Innocenti et al., (2000)
9	Dervieux 2001	Retrospective 78 patients, pediatric, European	6-MP 50 mg/m2/d > 5 months	Methotrexate	TPMT	6-TGN	RBC	173–1334 (pmol/8 × 108 RBC)	As steady-state 6-TGN concentrations became higher, leukocyte counts turned lower, the former and latter were significantly related	Dervieux et al., (2001) (Dervieux et al., 2001)
10	Stoneham 2003	Retrospective 99 patients, pediatric, European	6-MP (37%) or 6-TG (63%) >1 year	—	—	6-TGN	RBC	without VOD: 1231–1979 with VOD: 1240–1965 (pmol/8 × 108 RBC)	Risk factors responsible for VOD included male sex and 6-TG.	Stoneham et al., (2003)
11	Nygaard 2004	Retrospective 43 patients, pediatric, European	6-MP 75 mg/m2/d > 4 weeks	Methotrexate	TPMT	6-MMPN	RBC	1843–12422 (nmol/mmol Hb)	Weighted means of aminotransferase levels were significantly related to the average doses of 6-MP, erythrocyte levels of 6-MMPN, and TPMT activity. Weighted means of aminotransferase levels were negatively correlated with the erythrocyte levels of 6-TGN and were not related to the average doses of methotrexate or erythrocyte levels of methotrexate and its polyglutamates	Nygaard et al., (2004)
						6-TGN		162–488 (nmol/mmol Hb)		
12	Halonen 2006	Prospective 16 patients, pediatric, European	6-MP 75 mg/m2/d 2.3 years (SR) 1.7 years (IR)	Methotrexate Steroids Vincristine	—	6-TGN	RBC	86–301 (nmol/mmol Hb)	Throughout the therapy period, serum median ALT levels related significantly in a positive manner to the cumulative doses of 6-MP, but this correlation was not found when it came to the cumulative doses of 6-TGN.	Halonen et al., (2006)
13	Lennard 2006	Prospective 744 patients, pediatric, European	6-MP 75 mg/m2/d > 7 days	—	TPMT	6-TGN	RBC	682–4,072 (pmol/8 × 108 RBC)	The range of 6-TGNs in either the persistent splenomegaly group or the splenomegaly and VOD cohort did not differ from the range recorded in control children taking 6-MP.	Lennard et al., (2006)
14	Ganping 2008	Prospective 10 patients, pediatric, Asian	6-MP 75 mg/m2/d > 2 months	Methotrexate	—	6-TGN	RBC	day 7: 264–866 days 14: 290–450 (pmol/8 × 108 RBC)	275–750 pmol/8 × 108/sup RBC was the established and recognized target level range of 6-TGN. Just by determining the level of 6-TGN in blood sample as well as modifying the 6-MP dosage in accordance with 6-TGN concentrations. It’s workable to achieve the individualization and personalization of 6-MP.	Ganping Zhou et al., (2008)
15	Adam de Beaumais 2011	Prospective 66 patients, pediatric, European	6-MP 50 mg/m2/d 15 months	Methotrexate Aracytine Steroids	TPMT	6-MMPN	RBC	TPMT WT: 11290 TPMT HT: 5,010 (pmol/8 × 108 RBC)	In terms of 6-TGN concentrations and hepatotoxicity, no strong relationship was determined between the two	[Adam de Beaumais et al., (2011)]
						6-TGN		TPMT WT: 336 TPMT HT: 757 (pmol/8 × 108 RBC)		
16	Wojtuszkiewicz 2014	Prospective 236 patients, pediatric, European	6-MP week 6–161	—	—	6-TGN	RBC	week 25: 38–934 weeks 53: 68–881 weeks 109: 84–1048 (pmol/8 × 108 RBC)	Greater *in vitro* antileukemic activity was demonstrated to tend to be associated with high levels of 6-TGN. And elevated 6-TGN concentrations were proved to be strongly related to grade III/IV leucopenia	Wojtuszkiewicz et al., (2014)
17	Moriyama 2017	Prospective 55 patients, pediatric, Asian	6-MP 50 mg/m2/d > 13 weeks	—	NUDT15	DNA-TG	WBC	78.1–1054.0 (fmol TG/μg DNA)	For NUDT15-deficient patients, the ratio of DNA-TG to TGN was dramatically raised; To judge the adjustments of NUDT15 genotype-guided dose, compared to TGN, DNA-TG is a more pertinent MP metabolite	Moriyama et al., (2017)
						6-TGN	RBC	0.46–315.5 (pmol/4 × 108 RBC)		
18	Nielsen 2017	Prospective 1266 patients, pediatric, European	6-MP 75 mg/m2/d TPMT heterozygous: 50 mg/m2/d TPMT-deficient: 10 mg/m2/d > 37 weeks	Methotrexate Steroids Vincristine	—	DNA-TG	WBC	phase 1: 23–1591 phase 2: 44–1559 (fmol TG/μg DNA)	DNA-TGN concentration was found to have significant correlation with relapse-free survival; elevated concentrations of DNA-TGN indicated raised relapse-free survival	Nielsen et al., (2017)
19	Gerbek 2018	Prospective 132 patients, pediatric, European	6-MP TPMTWT: 75 mg/m2/24 h	Methotrexate Steroids Vincristine	TPMT ITPA	6-TGN	RBC	178–305 (nmol/mmol Hb)	When wild-type patients were used as the baseline, in low-activity patients it was found their median DNA-TG levels were higher in TPMT and ITPA.	Gerbek et al., (2018)
						6-MMPN		9787–22233 (nmol/mmol Hb)		
						DNA-TG	WBC	272–458 (fmol TG/μg DNA)		
20	Choi 2019	Retrospective 139 patients, Pediatric, Asian	6-MP 50 mg/m2/d > 1 month	Methotrexate Steroids Vincristine Cytarabine Hydrocortisone	TPMT NUDT15 ITPA MRP4	6-TGN	RBC	301.1–555.2 (pmol/4 × 108 RBC)	The levels of thiopurine metabolites (6-TGN and 6-MMPN) were significantly associated with 6-MP dosage	Choi et al., (2019)
21	Ju 2021	Prospective 71 patients, pediatric, Asian	6-MP 50 mg/m2/d > 2 weeks	—	NUDT15 TPMT	DNA-TG	WBC	1.0–903.1 (fmol TG/μg DNA)	During the time when patients suffered from the leukopenia episodes, the DNA-TGN concentrations varied between 27.8 and 54.8 fmol TG/μg DNA.	Ju et al., (2021)
22	Larsen 2021	Prospective 52 patients, pediatric, European	6-MP	Methotrexate Steroids Vincristine	—	DNA-TG	WBC	31–2888 (fmol TG/μg DNA)	A dependable profile of DNA-TG levels could be offered by measuring DNA-TG at 2–4 week intervals	Larsen et al., (2021a)
23	Larsen 2021	Prospective 34 patients, pediatric and adult European	6-MP+6-TG >10 weeks	Methotrexate	—	DNA-TG	WBC	764 (mean) (fmol TG/μg DNA)	It is a novel and practicable method to add incremental doses of 6-thioguanine to methotrexate/6-mercaptopurine, maintenance therapy, which can enhance the therapy, and consequently boost greater DNA-TG with no extra toxicity induced	Larsen et al., (2021b)
24	Nielsen 2021	Retrospective 918 patients, pediatric, European	6-MP TPMT heterozygous: 50 mg/m2/d	Methotrexate Steroids Vincristine Asparaginase	TPMT	DNA-TG	WBC	wildtype: 492.7 heterozygous: 760.9 (fmol TG/μg DNA)	TPMT heterozygous patients had higher DNA-TG levels	Nielsen et al., (2021)
25	Rosdiana 2021	Cross-sectional 106 patients, pediatric, Asian	6-MP 50 mg/m2/d > 1 month	Methotrexate Steroids Vincristine	TPMT	6-MMPN	RBC	3.5–3167.01 (pmol/8 × 108 RBC)	6-MMPN plasma concentrations and 6-MMPN/6-TGN ratio were found to be linked with the occurrence of hematotoxicity	Rosdiana et al., (2021)
						6-TGN		6–234.04 (pmol/8 × 108 RBC)		
26	Toksvang 2021	Prospective 1234 patients, pediatric and adult, European	6-MP 75 mg/m2/d TMPT heterozygous: 50 mg/m2/d	Methotrexate PegASP Steroids	TPMT	6-MMPN	RBC	0–103323 (nmol/mmol Hb)	By determining and judging 6 MP and MTX metabolites, the intensity of maintenance therapy could be ascertained, and it had no relationship with the danger of getting osteonecrosis	Toksvang et al., (2021)
						6-TGN		0–5,966 (nmol/mmol Hb)		
						DNA-TG	WBC	30–5,610 (fmol TG/μg DNA)		
27	Fan, P. 2022	Retrospective 145 patients, pediatric, Asian	6-MP 50 mg/m2/d > 6 months	Methotrexate	TPMT NUDT15 ITPA MRP4	DNA-TG	WBC	246.5 ± 267.8 (fmol TG/μg DNA)	A significantly higher DNA-TG to dose ratio was indicated in the patients who experienced one or more leukopenia episodes	Fan et al., (2022)

ADR, adverse drug reaction; PegASP, Pegylated-asparaginase; GI, symptoms: gastrointestinal symptoms; VOD, vena-occlusive disease; SR, standard risk; IR, intermediate risk; WT, wild-type; HT, carrier of one variant allele; 6-MP, 6-mercaptopurine; TPMT, thiopurine S-methyl transferase; NUDT15, Nudix hydrolase 15; 6-TGN, 6-thioguanine nucleotides; 6-MMPN, methyl-thioIMP; DNA-TG, DNA-incorporated thioguanine; ITPA, inosine triphosphate pyrophosphatase; RBC, red blood cells; WBC, white blood cells.

### Concentrations of 6-TGNs in RBCs is related to neutropenia and relapsed risk

In ALL patients, the 6-TGN concentrations in RBCs have been recognized to correlate with neutropenia and tolerable 6-MP doses in the 1980s (Lennard et al., 1983; Lilleyman et al., 1984; Lennard and Lilleyman, 1989), whereas the well-established reference range of 6-TGN therapeutic levels has not yet been agreed (Schmiegelow and Bruunshuus, 1990; Lancaster et al., 1998; Innocenti et al., 2000; Stoneham et al., 2003; Halonen et al., 2006; Lennard et al., 2006). A study by Chrzanowska et al. showed the 6-TGN concentrations that ranged from <60 to 833 pmol/8 × 10^8^ RBC in patients receiving 6-MP dosed at 50 mg/m^2^/day (Chrzanowska et al., 1999). In the study by Bhatia et al., patients received a higher dose of 6-MP, 75 mg/m^2^/day and the 6-TGN levels remained 0.3–714.1 pmol/8 × 10^8^ RBC (Bhatia et al., 2015). Nevertheless, the levels obtained in the study by Rosdiana et al. were 6–234.04 pmol/8 × 10^8^ RBC with a 6-MP dose of 50 mg/m^2^/day (Rosdiana et al., 2021). Interestingly, Zhou Y and others proposed the existence of a target threshold of 197.50 pmol/8 × 10^8^ RBCs to predict the risk of leukopenia in Chinese pediatric patients tormented by ALL (Zhou et al., 2020). Notably, not all studies found a correlation between metabolite concentrations and adverse effects (Halonen et al., 2006), which may due to the different therapy regimens (Lancaster et al., 1998; Lennard et al., 2006). More importantly, 6-TGN concentrations were associated with relapsed risk (Lennard et al., 1990; Wojtuszkiewicz et al., 2014). Therefore, there is an urgent to conduct a multicenter study involving more patients to find a suitable target range of therapeutic targets.

### Ratio of 6-MMPN/6-TGN is associated with the efficacy and tolerance of thiopurines

As previously described, a clear correlation between 6-MMPN concentrations and the development of hepatotoxicity was found in ALL patients treated with 6-MP (Nygaard et al., 2004; Adam de Beaumais et al., 2011). The findings of Nygaard et al. (Nygaard et al., 2004) indicated that the 6-MMPN contents were the most important pharmacological determinants of elevated aminotransferase levels during the 6-MP maintenance therapy in childhood ALL. A later study of Beaumais et al. showed that the threshold concentration of 6-MMPN, 4,884 pmol/8 × 10^8^ RBC could predict the risk of hepatotoxicity with a positive predictive value of 95.7% (Adam de Beaumais et al., 2011). As well known, patients carrying *TPMT* mutations had higher TGN levels than their wild-type counterparts, but this genetic variation only interprets the intolerance in 30–60% patients who received full doses of 6-MP or AZA (Relling et al., 2011). Some heterozygotes might be sufficiently thiopurine-tolerant because they have lower 6-MMPN concentrations than those with homozygous wild-type carriers (and thus fewer toxic effects), and therefore tolerated higher 6-TGNs. For example, in Dervieux’s study, wild-type patients experienced higher 6-MMPN concentrations (median: 6,137 pmol/8 × 10^8^ cells) than those carrying *TPMT* mutations (median: 307 pmol/8 × 10^8^ cells) (Dervieux et al., 2001). In this study, the 6-MMPN concentrations in RBCs were determined in the last patients enrolled in the trail, with a median concentration of 5,749 pmol/8 × 10^8^ cells (range 20–19682). The authors confirmed that higher 6-MP dosage and infectious events were associated with higher 6-MMPN concentrations. More recently, studies have shown a link between the ratio of 6-MMPN/6-TGN and the incidence of grade 3–4 neutropenia (Rosdiana et al., 2021). The researchers therefore proposed that the balance between RBC 6-MMPN and 6-TGN levels was important for predicting efficacy and improving the tolerance of thiopurines. Unfortunately, the therapeutic ratio of 6-MMPN/6-TGN is also unclear up to now.

### DNA-incorporated thioguanine nucleotides and relapse-free survival during ALL maintenance therapy

Pharmacologically, leukocyte DNA-TG, a cytotoxic agent and the end-point metabolite of 6-MP (Karran and Attard, 2008), appears to be more appropriately and accurately reflecting therapy intensity in the nucleated target cells in ALL, and it has recently been associated with 6-TGNs and 6-MMPN in RBCs (Hedeland et al., 2010; Ebbesen et al., 2013; Vang et al., 2015). Moreover, using an on-therapy blood count model, Nielsen and others attempted to estimate the degree of myelosuppression using the DNA-TG levels, a parameter available on-therapy that could be indicative of the treatment intensity. However, the results were contradictory. On-therapy ANC decreased with increasing DNA-TGN level (*p* < 0.001, model adjusted for off-therapy ANC), whereas on-therapy absolute lymphocyte counts (ALC) could not be modeled reliably (Nielsen et al., 2016). The authors claimed that measurements of the DNA-TG levels could provide blood counts when evaluating therapy intensity, but required prospective validation. Subsequently, a prospective sub-study of a phase 3 trial (NOPHO ALL 2008) has confirmed that the DNA-TG concentration (adjusted hazard ratio 0·81 per 100 fmol/μg DNA increase, 95%CI 0.67–0.98; *p* = 0.029) was closely related to relapse-free survival (Nielsen et al., 2017). Although higher DNA-TG was associated with a decreased relapse rate, it was worth noting that the cytotoxicity of maintenance therapy in ALL was also dependent on DNA-TG formation. Larsen and others added a low-dose 6-TG to MTX/6 MP maintenance therapy, termed as “TEAM” strategy, which proved to be a novel and practicable method to enhance the maintenance therapy, resulting in higher DNA-TGs without causing additional toxicity (Larsen et al., 2021b). They recommended that a reliable profile of DNA-TG levels could be provided by measuring DNA-TGs in leucocytes every 2–4 weeks (Larsen et al., 2021a). On the other hand, as pharmacogenomics significantly influence the metabolism of 6-MP, numerous studies have investigated the influence of genotypes on the DNA-TG levels. In Gerbek’s study, Gerbek found that *TPMT* heterozygous patients held notably higher DNA-TG levels than *TPMT* wild-type carriers (Gerbek et al., 2018). Another study of Nielsen was consistent with those results (Nielsen et al., 2021). Gerbek and others also found that the DNA-TG levels were significantly elevated in ITPA heterozygotes compared with *ITPA* wild-type carriers (Gerbek et al., 2018). In addition, DNA-TG accumulated with higher efficiency *in vivo* as the amount of risk alleles increased in the *NUDT15* gene (Moriyama et al., 2017).

We speculate that those large inter-individual variations in previous studies might be caused by variations in 6-MP absorption and disposition, drug–drug interactions, different TPMT enzyme activities, ethnic differences, and by patients’ compliance. In addition, the stratification according to those risk factors could be performed, but many chemotherapies are still used in the same way when treating all types of ALL. Hence, as optimal metabolite levels may vary by indications, it is important for physicians to adapt posology so that toxicity can be reduced while efficacy is not affected.

## Conclusion and outlook

Thiopurines have been broadly used for over 5 decades in the treatment of a wide range of diseases. However, side effects for thiopurines ranging from mild rashes, flu-like symptoms to severe life-threatening myelosuppression, and hepatotoxicity have hindered their clinical application. Although pharmacogenetics and TDM for thiopurines may contribute to and influence clinical practice, there are still several problems that need to be addressed urgently.

Although the pharmacogenetics of thiopurines is one of the most successful clinical applications, the large intra- and inter-individual variations of thiopurines, especially the high incidence of side effects in Asian populations, remain difficult to explain well. One possible explanation is that, thiopurine-related toxicity phenotype could not be determined only by one or two genotyping analyses (e.g. *TPMT* and *NUDT15*). Conversely, while high-throughput techniques allow researchers to map thousands of genetic polymorphisms in a single test, it still remains an enormous challenge to identify and figure out which one exerts the most considerable impact on both efficacy and toxicity. We now propose in this review, that a comprehensive consideration of the “MINT” sequencing strategy including *MRP4*, *ITPA*, *NUDT15*, and *TPMT* genes, combined with patients’ clinical characteristics, will provide more accurate information for the precise medication of thiopurines. Furthermore, if rare SNPs could dramatically alter the properties of transporter proteins or enzymes in victims with thiopurine-related side effects, it would be beneficial to continue further research. Unfortunately, we still have a long way to go before incorporating pharmacogenetic tests into routine clinical practice, which can help predict the outcomes and effects of thiopurine therapy.

For TDM of active metabolites, the therapeutic levels of 6-TGNs, 6-MMPN, or their cut-offs remain inconsistent. Therefore, in clinical practice, physicians should be cautious about the calculated optimal threshold for adverse events. On the other hand, most studies measure the level of metabolites in RBCs to evaluate the effect of drug treatment, which are not representative of the drugs in lymphocytes. This may be the reason why various studies have come to different conclusions. Recent studies have found that the levels of DNA-TG may be more related to the clinical efficacy and adverse reactions of thiopurines, which is the development direction of TDM monitoring in the future. The standardization of procedures for the evaluation of metabolites should be attached great importance in the near future.

In conclusion, we propose that integrating the “MINT” sequencing strategy with routine DNA-TG- and 6-MMPN-monitoring might be more feasible toward improving the efficacy and tolerability of thiopurines. Nevertheless, multicenter studies with large samples in different ethnic populations need to be performed in the future.
