# Supplementary material for: Optimizing thiopurine therapy in children with acute lymphoblastic leukemia: A promising “MINT” sequencing strategy and therapeutic “DNA-TG” monitoring
Source: Front Pharmacol. 2022 Sep 27;13:941182. doi: 10.3389/fphar.2022.941182 (PMC9552076; doi:10.3389/fphar.2022.941182)
Supplement: Supplementary file 1 [file Table1.docx]

Table S1 Summary of effects of *TPMT*, *NUDT15*, *ITPA* and *MRP4* polymorphisms on clinical outcomes of thiopurines in ALL pediatric patients

| **Gene** | **Polymorphism(s)** | **Results** | **Ref.** |
| --- | --- | --- | --- |
| *TPMT* | *TPMT *2*, **3A*, **3B*, **3C* | - There was no significant difference between the groups with and without mutations. | [[1](#_ENREF_1), [2](#_ENREF_2)] |
|  |  | - *TPMT *1/*3C*, **1/*2*, **1/*3A* and **3A/*3C* were associated with decreased dose of mercaptopurine or thioguanine. | [[3-13](#_ENREF_3)] |
|  |  | - Patients with *TPMT *2*, **3A*, **3B* and **3C*) were at greater risk of hematological toxicity and death. | [[2](#_ENREF_2), [4](#_ENREF_4), [6](#_ENREF_6), [7](#_ENREF_7), [10-12](#_ENREF_10), [14-20](#_ENREF_14)] |
|  |  | - *TPMT *1/*3A*, **1/*3C* and **3A/*3A* were associated with increased discontinuation of mercaptopurine. | [[8](#_ENREF_8)] |
|  | *TPMT *12* | - *TPMT *12* was associated with increased severity of mercaptopurine-induced toxicity. | [[21](#_ENREF_21)] |
| *NUDT15* | *NUDT15 *2*, **3*. **4*, **5*, **6* | - *NUDT15 *2* and **3* had no correlation with event-free survival when treated with mercaptopurine. | [[22](#_ENREF_22), [23](#_ENREF_23)] |
|  |  | - *NUDT15 *2*, **3*, **4*, **5*, **6* were associated with decreased dose of mercaptopurine. | [[5](#_ENREF_5), [9](#_ENREF_9), [11](#_ENREF_11), [22-33](#_ENREF_22)] |
|  |  | - *NUDT15 *3* was associated with hepatotoxicity induced by mercaptopurine. | [[22](#_ENREF_22), [34](#_ENREF_34), [35](#_ENREF_35)] |
|  |  | - Patients with *NUDT15 *2*, **3*, **4* and **6* were at increased risk of hematological toxicity. | [[9](#_ENREF_9), [11](#_ENREF_11), [22](#_ENREF_22), [26](#_ENREF_26), [31](#_ENREF_31), [33](#_ENREF_33), [36-39](#_ENREF_36)] |
|  |  | - Patients with homozygous or heterozygous *NUDT15*3* had a reduced tolerance to mercaptopurine. | [[23](#_ENREF_23), [33](#_ENREF_33)] |
|  | rs73189762 (G > A/T) | - Allele T was associated with decreased dose of mercaptopurine. | [[40](#_ENREF_40)] |
| *ITPA* | rs1127354 (C > A/G) | - Genotype CC was not associated with dose of mercaptopurine. | [[26](#_ENREF_26), [41](#_ENREF_41)] |
|  |  | - Genotype CC had no correlation with risk of myelosuppression when treated with mercaptopurine. | [[26](#_ENREF_26)] |
|  |  | - Allele A was not associated with decreased dose of mercaptopurine. | [[9](#_ENREF_9), [13](#_ENREF_13)] |
|  |  | - Allele A was not associated with increased likelihood of mercaptopurine-induced hematological toxicity. | [[13](#_ENREF_13), [31](#_ENREF_31), [42](#_ENREF_42)] |
|  |  | - Allele A was associated with decreased dose of mercaptopurine | [[43](#_ENREF_43), [44](#_ENREF_44)] |
|  |  | - Patients with allele A were more likely to develop toxicity with 6-MP. | [[35](#_ENREF_35), [43](#_ENREF_43), [45](#_ENREF_45), [46](#_ENREF_46)] |
|  |  | - Genotype CC was associated with increased event free survival when treated with mercaptopurine. | [[47](#_ENREF_47)] |
|  |  | - Patients with genotype CC had increased risk of hematological toxicity when treated with mercaptopurine. | [[48](#_ENREF_48)] |
|  |  | - *ITPA* c.94C > A variant showed significant positive association with 6-MP intolerance. | [[33](#_ENREF_33)] |
|  |  | - Genotypes AA and AC were associated with increased severity of mercaptopurine-induced myelosuppression. | [[36](#_ENREF_36)] |
|  | rs7270101 (A > C) | - Genotype AA was not associated with dose of mercaptopurine. | [[41](#_ENREF_41)] |
|  |  | - Genotypes AC and CC were associated with increased likelihood of hematological toxicity when treated with azathioprine or mercaptopurine. | [[20](#_ENREF_20), [46](#_ENREF_46), [48](#_ENREF_48)] |
| *MRP4* | rs3765534 (C > T/G > A) | - Genotype CC was not associated with dose of mercaptopurine. | [[41](#_ENREF_41)] |
|  |  | - Genotype TT was associated with decreased dose of mercaptopurine. | [[49](#_ENREF_49)] |
|  |  | - Alleles T and A had a correlation with increased likelihood of hematological toxicity when treated with mercaptopurine. | [[31](#_ENREF_31), [33](#_ENREF_33)] |
|  | rs2274407 (C > A/G/T and G > A/C/T) | - Allele A was not associated with increased likelihood of mercaptopurine-induced hematological toxicity. | [[31](#_ENREF_31)] |
|  |  | - Genotype AA was associated with decreased dose of mercaptopurine. | [[49](#_ENREF_49)] |
|  |  | - Genotypes AA and AC were associated with decreased likelihood of event-free survival. | [[50](#_ENREF_50)] |
|  |  | - Genotype CC was associated with increased risk of hematological toxicity when treated with mercaptopurine. | [[48](#_ENREF_48)] |
|  |  | - Genotype AC was associated with increased severity of thrombocytopenia when treated with mercaptopurine as compared to genotype CC. | [[51](#_ENREF_51)] |
|  |  | - Genotypes GT and TT were associated with worse 3-year disease-free survival. | [[50](#_ENREF_50)] |
|  |  | - *MRP4* C > A mutation was associated with thiopurine-related toxicities. | [[52](#_ENREF_52)] |
|  |  | - *MRP4* c.912G > T variant showed significant positive association with 6-MP intolerance. | [[33](#_ENREF_33)] |

**Reference:**

1. Silva, M.R., et al., *Thiopurine S-methyltransferase (TPMT) gene polymorphism in Brazilian children with acute lymphoblastic leukemia: association with clinical and laboratory data.* Ther Drug Monit, 2008. **30**(6): p. 700-4.

2. McLeod, H.L., et al., *Analysis of thiopurine methyltransferase variant alleles in childhood acute lymphoblastic leukaemia.* Br J Haematol, 1999. **105**(3): p. 696-700.

3. Kapoor, G., et al., *Thiopurine S-methyltransferase gene polymorphism and 6-mercaptopurine dose intensity in Indian children with acute lymphoblastic leukemia.* Leuk Res, 2010. **34**(8): p. 1023-6.

4. Lennard, L., et al., *Thiopurine dose intensity and treatment outcome in childhood lymphoblastic leukaemia: the influence of thiopurine methyltransferase pharmacogenetics.* Br J Haematol, 2015. **169**(2): p. 228-40.

5. Zgheib, N.K., et al., *NUDT15 and TPMT genetic polymorphisms are related to 6-mercaptopurine intolerance in children treated for acute lymphoblastic leukemia at the Children's Cancer Center of Lebanon.* Pediatr Blood Cancer, 2017. **64**(1): p. 146-150.

6. Dokmanovic, L., et al., *Analysis of thiopurine S-methyltransferase polymorphism in the population of Serbia and Montenegro and mercaptopurine therapy tolerance in childhood acute lymphoblastic leukemia.* Ther Drug Monit, 2006. **28**(6): p. 800-6.

7. Andersen, J.B., et al., *Pharmacokinetics, dose adjustments, and 6-mercaptopurine/methotrexate drug interactions in two patients with thiopurine methyltransferase deficiency.* Acta Paediatr, 1998. **87**(1): p. 108-11.

8. Peregud-Pogorzelski, J., et al., *Thiopurine S-methyltransferase (TPMT) polymorphisms in children with acute lymphoblastic leukemia, and the need for reduction or cessation of 6-mercaptopurine doses during maintenance therapy: the Polish multicenter analysis.* Pediatr Blood Cancer, 2011. **57**(4): p. 578-82.

9. Zhou, H., et al., *Optimal predictor for 6-mercaptopurine intolerance in Chinese children with acute lymphoblastic leukemia: NUDT15, TPMT, or ITPA genetic variants?* BMC Cancer, 2018. **18**(1): p. 516.

10. Kham, S.K., et al., *Thiopurine methyltransferase polymorphisms in a multiracial asian population and children with acute lymphoblastic leukemia.* J Pediatr Hematol Oncol, 2002. **24**(5): p. 353-9.

11. Soler, A.M., et al., *TPMT and NUDT15 genes are both related to mercaptopurine intolerance in acute lymphoblastic leukaemia patients from Uruguay.* Br J Haematol, 2018. **181**(2): p. 252-255.

12. Karas-Kuzelicki, N., et al., *Heterozygosity at the TPMT gene locus, augmented by mutated MTHFR gene, predisposes to 6-MP related toxicities in childhood ALL patients.* Leukemia, 2009. **23**(5): p. 971-4.

13. Farfan, M.J., et al., *Prevalence of TPMT and ITPA gene polymorphisms and effect on mercaptopurine dosage in Chilean children with acute lymphoblastic leukemia.* BMC Cancer, 2014. **14**: p. 299.

14. McBride, K.L., et al., *Severe 6-thioguanine-induced marrow aplasia in a child with acute lymphoblastic leukemia and inherited thiopurine methyltransferase deficiency.* J Pediatr Hematol Oncol, 2000. **22**(5): p. 441-5.

15. Cardoso de Carvalho, D., et al., *Association between the TPMT*3C (rs1142345) Polymorphism and the Risk of Death in the Treatment of Acute Lymphoblastic Leukemia in Children from the Brazilian Amazon Region.* Genes (Basel), 2020. **11**(10).

16. Smid, A., et al., *PACSIN2 polymorphism is associated with thiopurine-induced hematological toxicity in children with acute lymphoblastic leukaemia undergoing maintenance therapy.* Sci Rep, 2016. **6**: p. 30244.

17. Tumer, T.B., et al., *The low frequency of defective TPMT alleles in Turkish population: a study on pediatric patients with acute lymphoblastic leukemia.* Am J Hematol, 2007. **82**(10): p. 906-10.

18. Ando, M., et al., *Genetic polymorphisms of thiopurine S-methyltransferase and 6-mercaptopurine toxicity in Japanese children with acute lymphoblastic leukaemia.* Pharmacogenetics, 2001. **11**(3): p. 269-73.

19. Alves, S., et al., *Thiopurine methyltransferase pharmacogenetics: alternative molecular diagnosis and preliminary data from Northern Portugal.* Pharmacogenetics, 1999. **9**(2): p. 257-61.

20. Hawwa, A.F., et al., *Pharmacogenomic studies of the anticancer and immunosuppressive thiopurines mercaptopurine and azathioprine.* Br J Clin Pharmacol, 2008. **66**(4): p. 517-28.

21. Dorababu, P., et al., *Genetic variants of thiopurine and folate metabolic pathways determine 6-MP-mediated hematological toxicity in childhood ALL.* Pharmacogenomics, 2012. **13**(9): p. 1001-8.

22. Tanaka, Y., et al., *Susceptibility to 6-MP toxicity conferred by a NUDT15 variant in Japanese children with acute lymphoblastic leukaemia.* Br J Haematol, 2015. **171**(1): p. 109-15.

23. Liang, D.C., et al., *NUDT15 gene polymorphism related to mercaptopurine intolerance in Taiwan Chinese children with acute lymphoblastic leukemia.* Pharmacogenomics J, 2016. **16**(6): p. 536-539.

24. Wong, F.C., et al., *NUDT15 variant and thiopurine-induced leukopenia in Hong Kong.* Hong Kong Med J, 2016. **22**(2): p. 185-7.

25. Moriyama, T., et al., *The effects of inherited NUDT15 polymorphisms on thiopurine active metabolites in Japanese children with acute lymphoblastic leukemia.* Pharmacogenet Genomics, 2017. **27**(6): p. 236-239.

26. Chiengthong, K., et al., *NUDT15 c.415C>T increases risk of 6-mercaptopurine induced myelosuppression during maintenance therapy in children with acute lymphoblastic leukemia.* Haematologica, 2016. **101**(1): p. e24-6.

27. Suzuki, H., et al., *Genotyping NUDT15 can predict the dose reduction of 6-MP for children with acute lymphoblastic leukemia especially at a preschool age.* J Hum Genet, 2016. **61**(9): p. 797-801.

28. Moriyama, T., et al., *Novel variants in NUDT15 and thiopurine intolerance in children with acute lymphoblastic leukemia from diverse ancestry.* Blood, 2017. **130**(10): p. 1209-1212.

29. Yang, J.J., et al., *Inherited NUDT15 variant is a genetic determinant of mercaptopurine intolerance in children with acute lymphoblastic leukemia.* J Clin Oncol, 2015. **33**(11): p. 1235-42.

30. Tsujimoto, S., et al., *Diplotype analysis of NUDT15 variants and 6-mercaptopurine sensitivity in pediatric lymphoid neoplasms.* Leukemia, 2018. **32**(12): p. 2710-2714.

31. Tanaka, Y., et al., *Interaction between NUDT15 and ABCC4 variants enhances intolerability of 6-mercaptopurine in Japanese patients with childhood acute lymphoblastic leukemia.* Pharmacogenomics J, 2018. **18**(2): p. 275-280.

32. Correa-Jimenez, O., et al., *Susceptibility to thiopurine toxicity by TPMT and NUDT15 variants in Colombian children with acute lymphoblastic leukemia.* Colomb Med (Cali), 2021. **52**(3): p. e2074569.

33. Fan, P.O.L., et al., *ABCC4, ITPA, NUDT15, TPMT and their interaction as genetic predictors of 6-mercaptopurine intolerance in chinese patients with acute lymphoblastic leukemia.* Pediatr Hematol Oncol, 2022. **39**(3): p. 254-266.

34. Wang, X. and W. Wang, *[Association between polymorphism of NUDT15 gene and hepatotoxicity induced by 6-MP in children with acute lymphoblastic leukemia].* Zhonghua Yi Xue Yi Chuan Xue Za Zhi, 2021. **38**(12): p. 1258-1261.

35. Chen, Z.Y., et al., *Association Between Genetic Polymorphisms of Metabolic Enzymes and Azathioprine-Induced Myelosuppression in 1,419 Chinese Patients: A Retrospective Study.* Front Pharmacol, 2021. **12**: p. 672769.

36. Pai, A.A., et al., *NUDT15 c.415C>T Polymorphism Predicts 6-MP Induced Early Myelotoxicity in Patients with Acute Lymphoblastic Leukemia Undergoing Maintenance Therapy.* Pharmgenomics Pers Med, 2021. **14**: p. 1303-1313.

37. Khaeso, K., et al., *NUDT15 is a key genetic factor for prediction of hematotoxicity in pediatric patients who received a standard low dosage regimen of 6-mercaptopurine.* Drug Metab Pharmacokinet, 2022. **43**: p. 100436.

38. Cao, M., et al., *Screening of Novel Pharmacogenetic Candidates for Mercaptopurine-Induced Toxicity in Patients With Acute Lymphoblastic Leukemia.* Front Pharmacol, 2020. **11**: p. 267.

39. Puangpetch, A., et al., *NUDT15 genetic variants are related to thiopurine-induced neutropenia in Thai children with acute lymphoblastic leukemia.* Pharmacogenomics, 2020. **21**(6): p. 403-410.

40. Moriyama, T., et al., *Comprehensive characterization of pharmacogenetic variants in TPMT and NUDT15 in children with acute lymphoblastic leukemia.* Pharmacogenet Genomics, 2022. **32**(2): p. 60-66.

41. Eldem, I., et al., *SLCO1B1 Polymorphisms are Associated With Drug Intolerance in Childhood Leukemia Maintenance Therapy.* J Pediatr Hematol Oncol, 2018. **40**(5): p. e289-e294.

42. De Ridder, L., et al., *Pharmacogenetics of thiopurine therapy in paediatric IBD patients.* Aliment Pharmacol Ther, 2006. **23**(8): p. 1137-41.

43. Khera, S., et al., *Prevalence of TPMT, ITPA and NUDT 15 genetic polymorphisms and their relation to 6MP toxicity in north Indian children with acute lymphoblastic leukemia.* Cancer Chemother Pharmacol, 2019. **83**(2): p. 341-348.

44. Moradveisi, B., et al., *ITPA, TPMT, and NUDT15 Genetic Polymorphisms Predict 6-Mercaptopurine Toxicity in Middle Eastern Children With Acute Lymphoblastic Leukemia.* Front Pharmacol, 2019. **10**: p. 916.

45. Wan Rosalina, W.R., et al., *Polymorphism of ITPA 94C>A and risk of adverse effects among patients with acute lymphoblastic leukaemia treated with 6-mercaptopurine.* J Clin Pharm Ther, 2012. **37**(2): p. 237-41.

46. Azimi, F., et al., *Frequency of ITPA gene polymorphisms in Iranian patients with acute lymphoblastic leukemia and prediction of its myelosuppressive effects.* Leuk Res, 2015. **39**(10): p. 1048-54.

47. Kim, H., et al., *Pharmacogenetic analysis of pediatric patients with acute lymphoblastic leukemia: a possible association between survival rate and ITPA polymorphism.* PLoS One, 2012. **7**(9): p. e45558.

48. Hareedy, M.S., et al., *Genetic variants in 6-mercaptopurine pathway as potential factors of hematological toxicity in acute lymphoblastic leukemia patients.* Pharmacogenomics, 2015. **16**(10): p. 1119-34.

49. Tanaka, Y., et al., *Multidrug resistance protein 4 (MRP4) polymorphisms impact the 6-mercaptopurine dose tolerance during maintenance therapy in Japanese childhood acute lymphoblastic leukemia.* Pharmacogenomics J, 2015. **15**(4): p. 380-4.

50. Mesrian Tanha, H., S. Rahgozar, and M. Mojtabavi Naeini, *ABCC4 functional SNP in the 3' splice acceptor site of exon 8 (G912T) is associated with unfavorable clinical outcome in children with acute lymphoblastic leukemia.* Cancer Chemother Pharmacol, 2017. **80**(1): p. 109-117.

51. Ansari, M., et al., *Polymorphisms in multidrug resistance-associated protein gene 4 is associated with outcome in childhood acute lymphoblastic leukemia.* Blood, 2009. **114**(7): p. 1383-6.

52. Choi, R., et al., *Pathway genes and metabolites in thiopurine therapy in Korean children with acute lymphoblastic leukaemia.* Br J Clin Pharmacol, 2019. **85**(7): p. 1585-1597.
